# Gimbal-Less Two-Axis Electromagnetic Microscanner with Twist Mechanism

**DOI:** 10.3390/mi9050219

**Published:** 2018-05-06

**Authors:** Yangkyu Park, Seunghwan Moon, Jaekwon Lee, Kwanghyun Kim, Sang-Jin Lee, Jong-Hyun Lee

**Affiliations:** School of Mechanical Engineering, Gwangju Institute of Science and Technology (GIST), Gwangju 61005, Korea; pyk@gist.ac.kr (Y.P.); msh@gist.ac.kr (S.M.); jkwon19@gist.ac.kr (J.L.); khinmf13@gist.ac.kr (K.K.); leessang@gist.ac.kr (S.-J.L.)

**Keywords:** gimbal-less, twist mechanism, electromagnetic, crosstalk, fast Fourier transform (FFT)

## Abstract

We present an electromagnetically driven microscanner based on a gimbal-less twist mechanism. In contrast to conventional microscanners using a gimbal-less leverage mechanism, our device utilizes a gimbal-less twist mechanism to increase the scan angle in optical applications requiring a large scanning mirror. The proposed gimbal-less scanner with twist mechanism increases the scan angle by 1.55 and 1.97 times for the slow and fast axes, respectively, under the same force; 3.64 and 1.97 times for the slow and fast axes, respectively, under the same maximum stress, compared to the gimbal-less leverage mechanism. The scanner with a 3-mm-diameter mirror and a current path composed of a single-turn coil was fabricated, and it showed the maximum scan angle of 5° (quasi-static) and 22° (resonant) for the slow and fast axes, respectively. The experimentally estimated crosstalk was as small as 0.47% and 0.97% for the fast and slow axes affected by the other axes, respectively, which was determined using a newly employed methodology based on fast Fourier transform.

## 1. Introduction

Microelectromechanical systems (MEMS)-based optical microscanners have been widely developed in various optical applications owing to their advantages such as low power consumption, high speed, compact size, and low production cost [1]. Furthermore, MEMS scanners can easily perform two-dimensional (2D) scans, which is one of the most important advantages over conventional macroscanners [2]. Such advantages have promoted the use of MEMS scanners in a variety of applications such as optical communications [3], displays [1,4,5], biomedical imaging [6], and light detection and ranging (LiDAR) [7,8]. 

Over the past decades, a number of actuation strategies such as electrostatic [9], piezoelectric [10], thermal [6,11], and electromagnetic mechanisms [1,2,5,8,12] have been developed to actuate a micromirror. Electrostatic actuation can provide fast response time, low power consumption, and size advantages. However, the need for high applied voltages to achieve a large scan angle remains an issue to be resolved [1]. Piezoelectric actuation can obtain high force densities using materials with large piezoelectric constants; however, the large scan angles are limited owing to the short stroke length [13]. Thermal actuation has a relatively slow response time, even though a large scan angle can be achieved at a low actuation voltage [1,13]. In contrast, electromagnetic actuation can provide relatively large torque and fast response time. This causes electromagnetic scanners to be preferable in applications requiring large scan angles and high speeds such as LiDAR.

Compared to a gimbaled structure requiring an additional frame for the slow axis, a gimbal-less structure generally shows a relatively high resonant frequency in the slow axis, such that a high-speed quasi-static scanning can be achieved in electrostatic scanners [9]. In addition, leverage mechanisms can enhance the scan range in quasi-static actuations without large vertical or angular offsets between the stationary and movable comb electrodes [9]. However, in optical applications requiring a large mirror, the expected scan angle would be small because a large distance between the hinge (called for rotation transformer) and mirror reduces the leverage amplification ratio.

Herein, the design, fabrication, and experimental characterization of a gimbal-less two-axis electromagnetic microscanner are presented. In contrast to previous gimbal-less scanners with a leverage mechanism, our gimbal-less scanner utilizes a twist mechanism to enhance the scan angle in optical applications requiring a large scanning mirror. The unique current path and magnet assembly are designed to maximize the torque at the active current path and nullify the torque at the passive current path. To verify the proposed two-axis actuation, the frequency response and optical scan angle of the fabricated scanner is experimentally examined. In particular, fast Fourier transforms (FFT) are employed to accurately evaluate crosstalk in the two axes. To the best of our knowledge, this is the first study to actuate a mirror using gimbal-less structure with twist mechanism, and the crosstalk analysis based on FFT has not been attempted for the performance evaluation of the microscanners. 

## 2. Design

### 2.1. Working Principle

**Figure 1:**
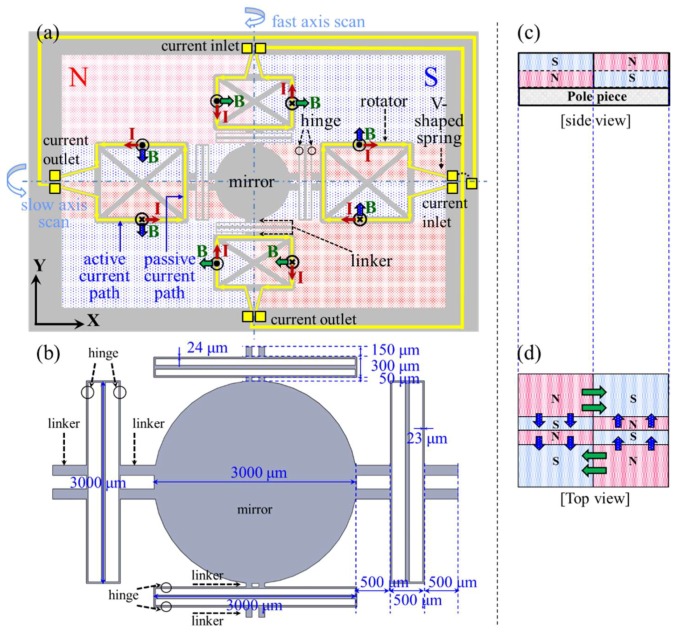
Schematics of the proposed scanner. (**a**) Top view of the scanner chip on magnet assembly; (**b**) dimensional figure for the hinges, linkers, and mirror; (**c**) side view of magnet assembly; (**d**) top view of magnet assembly.

Figure 1a,b shows the schematic top view of the proposed gimbal-less scanner with twist mechanism. A mirror is connected to the hinges by linkers consisting of dual parallel rigid bars. The hinges are composed of dual parallel flexible bars, which are capable of torsion and the first mode of in-plane-bending to facilitate two-axis actuations. The rotators connected to the hinges by the linkers are supported by a fixed frame through the torsional springs. The V-shaped torsional springs were employed to reduce the in-plane rolling of the movable parts [14] and to effectively arrange the current path. 

In general, the microcoils in electromagnetic scanners are composed of an active current path actuating a mirror and a passive current path completing an electrical current loop. As depicted in Figure 1, the rectangular-shaped current path in our proposed device allows the active and passive current paths to be orthogonal and parallel to each corresponding magnetic field, respectively. This can maximize the electromagnetic force at the active current path, while nullifying the force at the passive current path. Further, the current path is electrically separated for an independent biaxial scan. Compared to the gimbaled structure whose current path for the fast-axis scan is formed in the vicinity of the mirror [2,4,8,12], the proposed current path is away from the mirror and near the fixed frame, which could minimize mirror deformation due to Joule heating.

**Figure 2:**
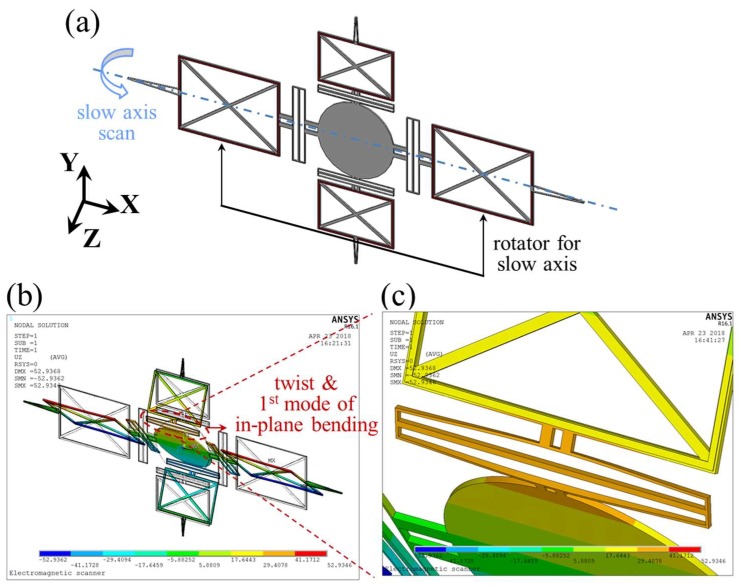
Finite element analysis (FEA) for driving characteristics. (**a**) Simulated model; (**b**) static displacement for slow-axis scan; (**c**) magnified view for the displacement of two hinges. The simulation was conducted under an applied force of 862.4 μN on each rotator for the slow axis.

The rotational direction of a mirror is determined by the direction of the current flow and magnetic field. A slow-axis scan is quasi-statically operated by the active current path on the rotators along the *x*-axis, which are orthogonal to the *y*-directional magnetic field (blue arrows in Figure 1). As shown in Figure 2a, when the rotators are actuated around the *x*-axis, the torsional springs connected to the rotators are twisted. Subsequently, each hinge along the *y*-axis is twisted and bent in the first mode of the in-plane-bending to reduce the torsional stiffness of the springs, facilitating actuation for the slow-axis scan (Figure 2b,c). The same kinematics as the slow-axis scan can be applied to the fast-axis scan. The fast-axis scan is resonantly operated by the active current path on the rotators along the *y*-axis, which are orthogonal to the *x*-directional magnetic field (green arrows in Figure 1). The calculated in-plane-bending stiffness are 71.46 N/m and 64.11 N/m for the slow and fast axis, respectively. 

### 2.2. Device Configuration

The gimbal-less structure does not require a gimbaled frame for the slow axis, thus achieving a higher resonant frequency than the gimbaled structure. Therefore, the gimbal-less structure can provide a relatively high speed and large bandwidth in the slow axis; furthermore, it can potentially be widely used in optical applications for not only the raster scan but also the Lissajous and vector scans. 

In gimbal-less leverage mechanism, a rotator and mirror are actuated around different rotation axes. This method has been commonly used in electrostatic gimbal-less scanners to increase the scan angle. The leverage amplification is determined by the ratio of the distance between the rotator and hinge to the distance between the hinge and mirror [9]. However, the leverage mechanism is not effective for large scanning mirrors, because the distance from the hinge to the mirror is large. Although the amplification can be enhanced by increasing the distance from the rotator to the hinge, the risks of large driving force and/or higher stress should be considered. In contrast, the gimbal-less twist mechanism is operated with the actuation of a rotator and mirror around the same rotation axis. Because this method does not involve leverage amplification, the scan angle is less affected by the mirror size.

A finite element analysis (FEA; ANSYS APDL, version R16.1, Ansys, Inc., Canonsburg, PA, USA) was performed to numerically compare the scan angle of the twist mechanism with the corresponding leverage mechanism under the identical dimensions in a mirror (3 mm in diameter), springs, rotators, hinges, and total device size. According to the simulation results, the scan angles of the twist mechanism were considerably increased by 1.55 and 1.97 times for the slow- and fast-axis scan, respectively, under the same force. The scan angles were also increased by 3.64 and 1.97 times for the slow- and fast-axis scan, respectively, under the same maximum stress. Thus, the gimbal-less twist mechanism can achieve a higher speed in the slow axis than the conventional gimbaled scanner, and provide a larger scanning angle with reduced stress than the conventional gimbal-less scanner.

### 2.3. Magnetic Field

The magnet assembly was designed to generate a rectangular-shaped magnetic field, which is appropriate for the current path of the proposed device. The magnet assembly whose area is the same as the scanner chip is attached at the bottom of the microscanner chip to provide a compact package. As shown in Figure 1c,d, the magnet assembly comprises eight (4 × 2) rectangular permanent magnets and a pole piece, providing independent magnetic fields for the fast- and slow-axis scans under the scanner chip.

**Figure 3:**
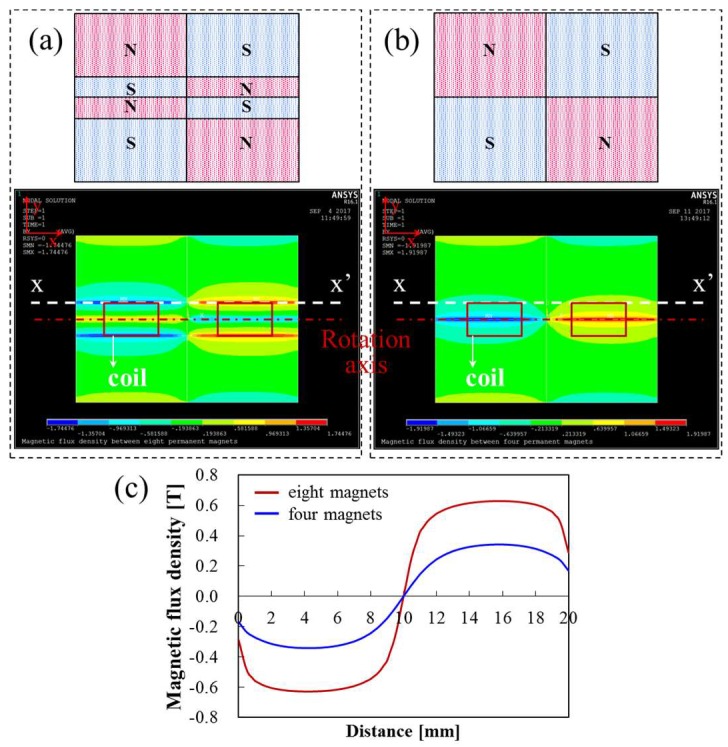
FEA results for *y*-directional magnetic flux density along the horizontal line (*xx*’) including active coils for slow-axis scan (0.45 mm above the surface of the magnet, 1.5 mm away from the rotational axis for slow-axis scan). (**a**) Active coil on the boundary between magnet poles (eight magnets); (**b**) active coil on the surface of magnets (four magnets); (**c**) comparison of *y*-directional magnetic flux density at the active current path depending on the type of magnetic assembly.

The designed magnet assembly was intended to provide the active current path for the slow-axis scan (quasi-static actuation) with a stronger magnetic field in the lateral direction rather than for the fast-axis scan (resonant actuation). Specifically, the active current path for the slow-axis scan was positioned on the boundary between the magnetic poles, using eight permanent magnets. As shown in Figure 3a,b, FEA was performed to numerically compare the magnetic flux density in two cases: the active coil on the boundary between the magnetic poles (eight magnets) and the active coil positioned on the magnet surface (four magnets) under the identical dimension as a whole. Figure 3c shows the *y*-directional magnetic flux density along the horizontal line (*xx*’) including the active current path for the slow axis (0.45 mm above the surface of the magnet, 1.5 mm away from the rotational axis for the slow-axis scan). According to the simulation results, the magnetic flux density of the eight magnets are approximately two times stronger than that of the four magnets, which indicates that the present magnetic design can maximize the electromagnetic force for quasi-static actuations. The active current path for the fast-axis scan (resonant actuation) can also be arranged on the boundary between the magnetic poles by modifying the present magnetic design. However, this leads to a decrease in the magnetic flux density for the slow-axis scan and even results in a complicated magnetic design.

## 3. Fabrication

The proposed scanner with a 3-mm-diameter mirror was fabricated using the double-sided etching process in a four-inch silicon-on-insulator (SOI) wafer. The thickness of the top (silicon), middle (oxide), and bottom (silicon) layers are 50, 2, and 400 μm, respectively. Via-less current path composed of single-turn microcoils was employed to simplify the fabrication sequence. 

**Figure 4:**
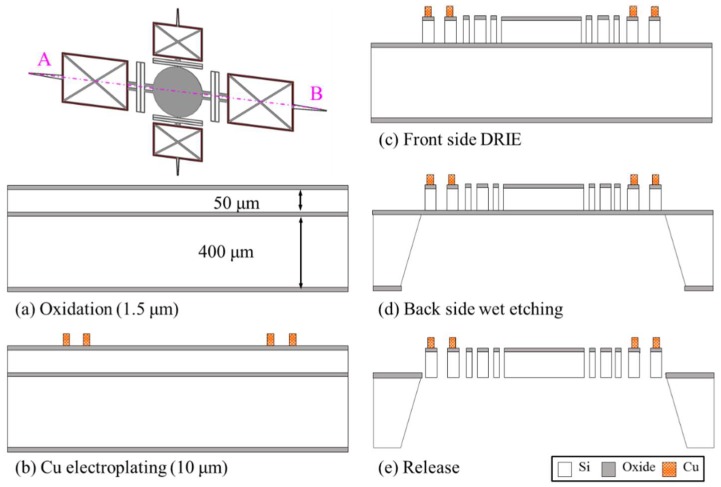
Fabrication process of the proposed scanner (cross-sectional images along line AB).

To fabricate the electrical parts (current path), the SOI wafer was initially oxidized in a furnace (Figure 4a). The thermal oxidation of the wafer was required for the insulation layer between copper and silicon. Subsequently, a 200-nm-thick seed layer (titanium and copper) was sputtered on the top layer and a 16-μm-thick photoresist (PR) was patterned by photolithography on the seed layer. During the thick-PR photolithography, the PR pattering for a wide electrical pad on the fixed frame required more time to be fully developed, compared to the narrow electrical line on the V-shaped torsional spring. This might cause the electrical line on the spring to become wider than the designed dimension. To overcome this problem, a grid structure in the electrical pad was applied to enhance the uniformity of the development time for the fabrication of the PR mold. Using the PR mold, a copper microcoil was electroplated to a thickness of 7 μm (Figure 4b).

To fabricate the mechanical parts (mirror, hinges, rotators, springs, and fixed frame), 16-μm-thick PR covering the copper on the top layer was patterned by photolithography. The patterned PR was used as a mask to selectively etch the thermal oxide in reactive ion etching (RIE). Next, the 50-μm-thick silicon of the top layer was completely etched through deep reactive ion etching (DRIE) until the middle layer (oxide) was exposed (Figure 4c). The electroplated copper was not damaged during the whole etching process because the metal layers were fully protected by the PR mask.

For the backside opening, the oxide layer on the bottom side was etched by RIE, using a 2 μm-thick PR mask formed by photolithography. Subsequently, the patterns of the thermal oxide were transferred to a 400-μm-thick bottom layer by wet etching, providing an opening space for mirror rotation (Figure 4d). Finally, the movable part was released by etching the middle layer (oxide), as shown in Figure 4e.

**Figure 5:**
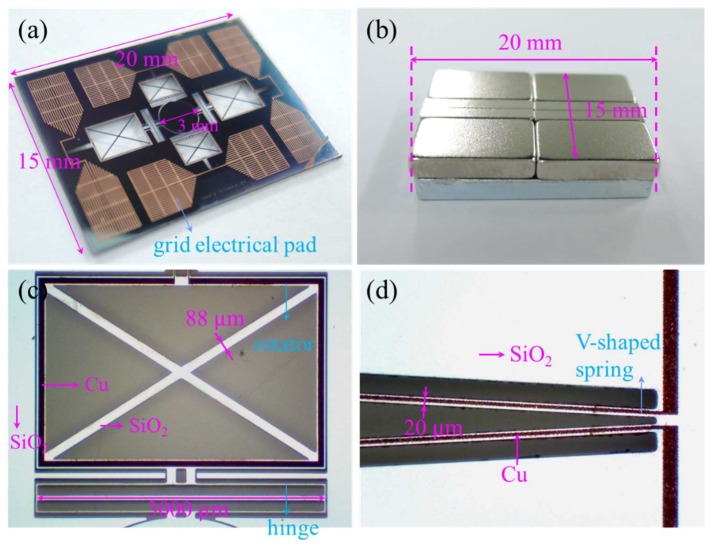
Photographs of the fabricated electromagnetic scanner. (**a**) Overall view of microscanner chip; (**b**) overall view of magnet assembly; (**c**) microscopic view of rotator and hinge; (**d**) microscopic view of V-shaped spring.

The magnet assembly was composed of eight Nd-Fe-B permanent magnets (KOMAGNET, Seoul, Korea) and a steel pole piece coated by electroplated zinc (HYOSUNG Mechanics, Gwangju, Korea). The scanner chip was bonded on the magnet assembly, using an adhesive glue epoxy (Cemedine, Tokyo, Japan). The magnetic assembly and scanner chip were fabricated on the same area to eliminate dead volume and to ease the alignment. The fabricated scanner chip and the magnet assembly are shown in Figure 5a,b, respectively. The total size of the integrated scanner was 15 mm × 20 mm × 4.46 mm. Figure 5c shows the microscopic images of the key components in the scanner chip including the rotator and hinge, while Figure 5d shows that of the V-shaped torsional spring.

## 4. Results and Discussion

### 4.1. Experimental Setup

**Figure 6:**
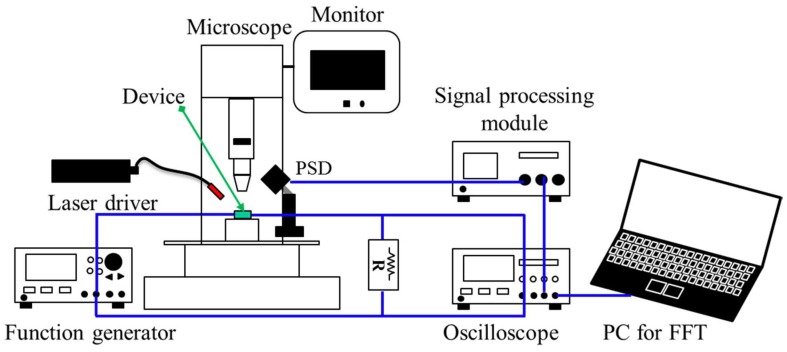
Experimental setup to characterize the fabricated scanner.

An experimental setup was prepared to characterize the proposed scanner, as shown in Figure 6. The driving signals were applied to the device through a function generator (AFG3102, Tektronix, Beaverton, OR, USA), and a collimator (LPC-01-633-4, OZ optics, Ottawa, ON, Canada) was used to emit a laser beam (λ = 633 nm) to the mirror at an angle of 45°. Subsequently, the reflected laser beam from the mirror was redirected toward a position-sensitive detector (PSD; PSD module C10460, Hamamatsu, Japan) at an incident angle of 90°. The position data from the PSD was obtained as output voltages, using an oscilloscope (DSO-X-4024A, Keysight, Santa Rosa, CA, USA). The applied current was measured through the voltage drop of a resistor (1 Ω) connected to the scanner in series. The PSD and scanner were precisely aligned to separate the scan angles along the fast and slow axes, minimizing geometrical crosstalk between the orthogonal scans. More specifically, during only one axis operation, the rotational stage under the PSD was finely adjusted until the scan angle of the other axis was minimized.

### 4.2. Frequency Response and Optical Scan Angle

**Figure 7:**
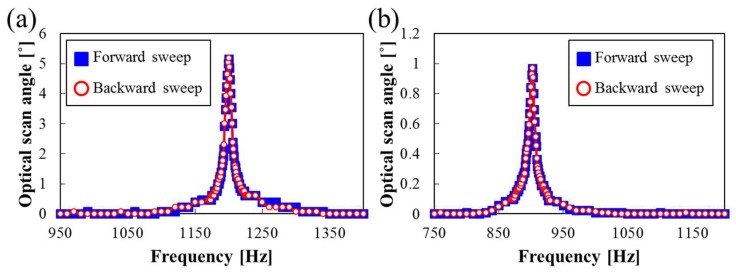
Frequency response in terms of driving frequency. (**a**) Slow axis; (**b**) fast axis. Blue squares and red circles represent the responses measured in forward and backward frequency sweep, respectively.

The experimental results of the frequency response for the slow- and fast-axis scans are shown in Figure 7. The frequency responses were experimentally measured with respect to the driving frequencies under atmospheric pressure with the sinusoidal input voltage set to 200 mV_pp_ for both axes. The resonant frequencies of the torsional mode were found to be 1200 Hz and 902 Hz for the slow- and fast-axis scans, respectively, in both forward and backward sweeps. To our best knowledge, the high resonant frequency of 1200 Hz in the slow axis has not been reported yet in microscanners with a mirror that has a diameter more than 3 mm. 

**Figure 8:**
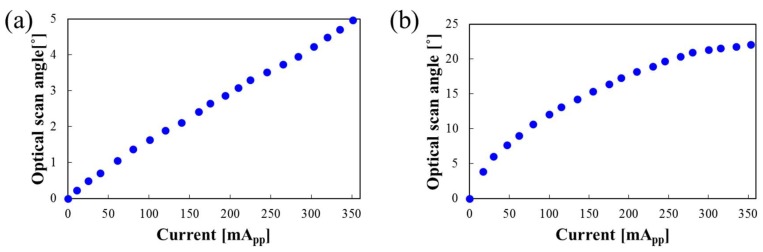
Optical scan angles with respect to the applied current. (**a**) Quasi-static actuation for the slow axis; (**b**) resonant actuation for the fast axis.

Figure 8 shows the optical scan angles with respect to the applied current. The optical scan angle for the slow-axis scan was measured under a quasi-static frequency of 60 Hz. The maximum scan angle of 5° was obtained at an applied current of 351 mA_pp_. The optical scan angle for the fast-axis scan was measured under a resonant actuation of 902 Hz. The maximum scan angle was 22° at the applied current of 353 mA_pp_. The voltages to provide the applied currents were 20 V_pp_ both for the slow and fast axes. It is noteworthy that this paper describes the feasibility of the gimbal-less twist mechanism. If our scanner is equipped with a current path composed of multi-turn coils, the device size can be reduced and the scan angle can be increased further. A follow-up design that takes into account current paths composed of multi-turn coils is currently being carried out to miniaturize a chip and improve device performance, which will be presented in a future paper.

### 4.3. Crosstalk Analysis

The crosstalk between the motions in two axes should be considered to determine the device performance. The crosstalk in conventional two-axis microscanners can be divided into two types: (1) electrical crosstalk due to the actuation of passive current path in a unidirectional magnetic field oriented 45° to the rotational axis [1,5] or the electrical coupling of the superimposed driving signal between two axes [4,5,8]; (2) mechanical crosstalk caused by the imperfection of the decoupling hinge. 

The electrical crosstalk can be minimized in the proposed scanner, because the passive current is parallel to the corresponding magnetic field and the current path is electrically separated, as aforementioned in Section 2. Although the coupled forces could be generated at the local passive current path owing to the formation of non-uniform magnetic fields, their forces are very weak and even counterbalanced with respect to each rotational axis, thus suppressing the electrical crosstalk between two axes. Meanwhile, the two axes of our scanner are mechanically decoupled using a flexible hinge. To ensure that the decoupling hinge can effectively prevent crosstalk between two axes, crosstalk should be experimentally investigated. 

In previous studies, to experimentally investigate crosstalk, the variation in the peak-to-peak value for the scan angle in one axis was measured in the time domain when the other axis was actuated [2,10]. However, in the time domain, the variation fluctuates because the electrical signal from the PSD includes not only actuation frequencies but also the *n*th harmonic components, thus hindering the accurate quantification of crosstalk. Therefore, crosstalk should be evaluated in the frequency domain using FFT processing. 

A methodology for the accurate evaluation of crosstalk is as follows: Initially, the device was operated together in the fast and slow axes at their maximum scan angles and corresponding frequencies (fast axis: 902 Hz, slow axis: 60 Hz). Subsequently, 62,500 data points for each scanning were sampled for 1 s from the oscilloscope (DSO-X-4024A, Keysight, Santa Rosa, CA, USA). Finally, the acquired data was converted from the time domain to the frequency domain with 1 Hz interval using FFT processing (MATLAB, version R2013b, MathWorks, Inc., Natick, MA, USA). 

**Figure 9:**
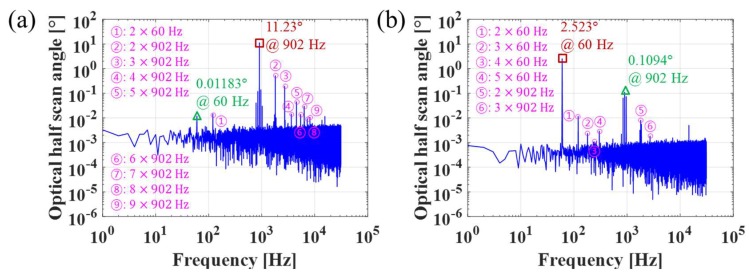
Crosstalk analysis in frequency domain. (**a**) Fast axis; (**b**) slow axis. The data was obtained when the scanner was oscillating with the maximum scan angles at 902 Hz and 60 Hz for the fast and slow axes, respectively. Rectangle (brown color), triangle (green color), and circle (pink color) represent actuation signal of one axis, coupled signal affected by the other axis, and the *n*th harmonics, respectively. Note that electrical noise at 60 Hz is negligible (0.1% of actuation signal at 60 Hz), which was verified by the FFT processing of data without slow-axis actuation.

Figure 9 shows the crosstalk analysis in the frequency domain (optical half scan angle versus frequency). In the fast axis as shown in Figure 9a, one peak value corresponding to 902 Hz (actuation signal in the fast axis) was the most dominant in the measurement frequency range. Another peak value corresponding to 60 Hz (coupled signal affected by the slow-axis actuation) was also observed in the fast axis. Additionally, although a sinusoidal input signal was applied to the device, the *n*th harmonic components (*n* × actuation frequency) were detected at several frequencies. More specifically, the harmonic signals at 1804, 2706, 3608, 4510, 5412, 6314, 7216, and 8118 Hz were induced by the fast-axis actuation at 902 Hz. The signal at 120 Hz is also thought to be induced by the slow-axis coupled frequency of 60 Hz. The harmonic signals appear to be attributed to the nonlinearity caused by the magnetic field variation and spring hardening effect with respect to the tilting angle. The slow axis showed a similar tendency to the fast axis, as shown in Figure 9b.

For accurate quantification, crosstalk in microscanners can be defined in Equations (1) and (2). The crosstalk in the fast axis affected by the slow-axis actuation (C_fs_) can be assessed through a calibration process in which the coupled signal in the fast axis (the scan angle influenced by the actuation frequency of slow axis in fast axis) is divided by the actuation signal in the slow axis (the scan angle in slow axis), as expressed by Equation (1). The same principle can be applied to the crosstalk in the slow axis affected by the fast-axis actuation (C_sf_), as shown by Equation (2).

(1) $$C_{\mathrm{fs}}\left[\%\right]=\frac{\mathrm{coupled}\;\mathrm{signal}\;\mathrm{in}\;\mathrm{fast}\;\mathrm{axis}}{\mathrm{actuation}\;\mathrm{signal}\;\mathrm{in}\;\mathrm{slow}\;\mathrm{axis}}\;\times \;100$$

(2) $$C_{\mathrm{sf}}\;\left[\%\right]=\;\frac{\mathrm{coupled}\;\mathrm{signal}\;\mathrm{in}\;\mathrm{slow}\;\mathrm{axis}}{\mathrm{actuation}\;\mathrm{signal}\;\mathrm{in}\;\mathrm{fast}\;\mathrm{axis}}\times \;100$$

The gimbal-less crosstalk of C_fs_ and C_sf_ were experimentally estimated as small as 0.47% and 0.97%, respectively. These values would be the possible maximum crosstalk, because the actuation condition is the worst in terms of crosstalk, considering that both axes are actuated at the maximum scan angles. Meanwhile, C_sf_ is larger than C_fs_, which can be explained by that the actuation frequency in the fast axis is close to the resonant frequency of the slow axis. We believe that crosstalk can be reduced if the resonance modes of two axes are separated further. Nevertheless, the proposed scanner substantially reduced the crosstalk to within 1%, compared to the previous gimbal-less microscanner [10].

## 5. Conclusions

A new type of gimbal-less two-axis electromagnetic scanner with twist mechanism has been designed, fabricated, and experimentally tested. A gimbal-less scanner can provide relatively high speed in the slow axis, compared to a gimbaled scanner. Our device utilizes gimbal-less twist mechanism instead of gimbal-less leverage mechanism to increase the scan angle for optical applications requiring a large size mirror. The fabricated scanner with a current path composed of a single-turn microcoil performed scan angles of 5° (quasi-static) and 22° (resonant) in the slow- and fast-axis scans, respectively. A methodology for the crosstalk measurement has been introduced; the crosstalk of the fast and slow axes affected by the other axis was measured as small as 0.47% and 0.97%, respectively. The proposed actuation principle of the device and measurement methodology of the crosstalk can be applied to various optical applications.
